# Trends in the Bacterial Prevalence and Antibiotic Resistance Patterns in the Acute Exacerbation of Chronic Obstructive Pulmonary Disease in Hospitalized Patients in South India

**DOI:** 10.3390/antibiotics11111577

**Published:** 2022-11-09

**Authors:** Mohammed Kaleem Ullah, Sowmya Malamardi, Jayaraj Biligere Siddaiah, Tejashree A, Akila Prashant, Prashant Vishwanath, Lee W. Riley, Purnima Madhivanan, Padukudru Anand Mahesh

**Affiliations:** 1 Centre for Excellence in Molecular Biology and Regenerative Medicine, Department of Biochemistry, JSS Medical College, JSS Academy of Higher Education & Research, Mysore 570015, Karnataka, India; 2 Division of Infectious Disease and Vaccinology, School of Public Health, University of California, Berkeley, CA 94720, USA; 3 Department of Respiratory Medicine, JSS Medical College, JSS Academy of Higher Education & Research, Mysuru 570015, Karnataka, India; 4 School of Psychology & Public Health, College of Science Health and Engineering, La Trobe University, Melbourne 3086, Australia; 5 Department of Microbiology, JSS Medical College, JSS Academy of Higher Education & Research, Mysuru 570015, Karnataka, India; 6 Department of Health Promotion Sciences, Mel & Enid Zuckerman College of Public Health, University of Arizona, Tucson, AZ 85724, USA; 7 Division of Infectious Diseases, College of Medicine, University of Arizona, Tucson, AZ 85724, USA; 8 Public Health Research Institute of India, Mysuru 570020, Karnataka, India

**Keywords:** pulmonary disease, chronic obstructive/complications, chronic obstructive/microbiology, bacterial infections/microbiology, drug resistance, multiple, bacterial, hospital mortality

## Abstract

Exacerbation due to antimicrobial-drug-resistant bacteria among chronic obstructive pulmonary disease (AECOPD) patients contributes to mortality and morbidity. We examined the prevalence of the bacterial organisms and trends in drug resistance in AECOPD. In this retrospective study, between January 2016 to December 2020, among 3027 AECOPD patients, 432 (14.3%) had bacteria isolated. The regression and generalized estimating equations (GEE) were used for trends in the resistance patterns over five years, adjusting for age, gender, and comorbidities. *Klebsiella pneumoniae* (32.4%), *Pseudomonas aeruginosa* (17.8%), *Acinetobacter baumannii* (14.4%), *Escherichia coli* (10.4%), and *Staphylococcus aureus* (2.5%) were common. We observed high levels of drug resistance in AECOPD patients admitted to ICU (87.8%) and non-ICU (86.5%). A Cox proportional hazard analysis, observed infection with *Acinetobacter baumannii* and female sex as independent predictors of mortality. *Acinetobacter baumannii* had 2.64 (95% confidence interval (CI): 1.08–6.43) higher odds of death, compared to *Klebsiella pneumoniae*. Females had 2.89 (95% CI: 1.47–5.70) higher odds of death, compared to males. A high proportion of bacterial AECOPD was due to drug-resistant bacteria. An increasing trend in drug resistance was observed among females.

## 1. Introduction

Chronic obstructive pulmonary disease (COPD) is a common, preventable, and treatable disease, characterized by persistent respiratory symptoms and an airflow limitation due to airway and/or alveolar abnormalities usually caused by exposure to noxious particles or gases [1]. The high burden of mortality from COPD is seen in Latin America, sub-Saharan Africa, India, China, and south-East Asia [2]. The prevalence of COPD in India varies from 2.5% to 10% [3,4]. An acute exacerbation of COPD (AECOPD) is defined as the acute worsening of respiratory symptoms in COPD patients that results in additional therapy [1]. COPD patients suffer one to four exacerbations per year, which accounts for an increased morbidity, mortality, and health-related expenditure [5]. COPD patients are immune deficient and have alterations in their lung microbiome that may result in a persistent bacterial infection, triggering acute exacerbations, and these infections are the most common cause of AECOPD, implicated in 40% to 60% of cases [6]. The clinical management of these infections, caused by drug-resistant bacteria becomes complicated. The frequency of the specific resistant bacteria causing AECOPD, varies according to the hospital, population of patients, exposure to antibiotics, and type of ICU patients. Globally, bacteria causing AECOPD vary, which commonly include *Haemophilus influenzae*, *Pseudomonas aeruginosa*, *Streptococcus pneumoniae*, and *Moraxella catarrhalis*.

Antibiotic resistance is rising to dangerously high levels in all parts of the world, threatening our ability to treat common infectious diseases [7]. Our armamentarium of effective antimicrobial drugs is declining rapidly [8], which has become a global public health threat. Antibiotics are the main form of treatment for AECOPD, which are often initiated empirically, based on healthcare providers’ previous experiences, which often lead to the inappropriate use of antibiotics, thereby contributing to the selection of antimicrobial resistance. There is a paucity of data on drug-resistant bacteria causing AECOPD, especially in countries, such as India, where antibiotic resistance is alarmingly increasing [9]. The widespread use of antibiotics has facilitated the selection and spread of multi-drug-resistant (MDR), extensively or extremely drug-resistant (XDR), and pan-drug-resistant (PDR) strains of these bacteria, which can render even the most effective drugs ineffective [10].

This paper describes a retrospective study conducted to identify the prevalence of bacterial species causing severe AECOPD and their trend in drug resistance patterns. The observations made in this study could help us develop tailored empirical antibiotic use strategies and prevent the emergence of drug resistance by improving our antimicrobial stewardship programs.

## 2. Results

**Figure 1:**
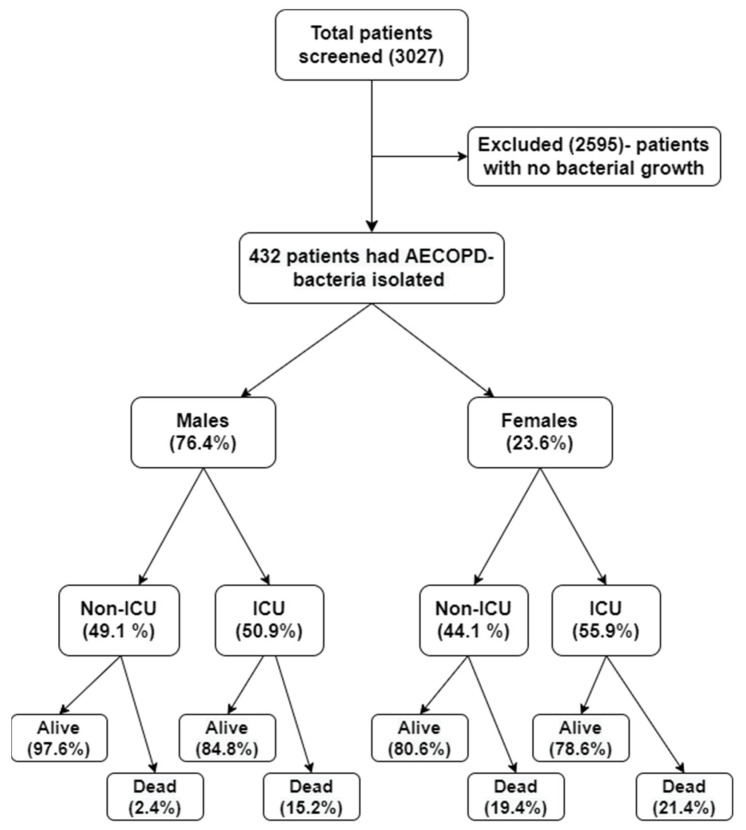
Flowchart of the study identifying the total number of subjects screened, number of subjects in whom bacteria have been isolated, their gender distribution, their place of admission (ICU or non-ICU), and the rates of in-hospital mortality.

Figure 1 shows 432 patients (14.3%) admitted with bacterial AECOPD, that were included in the study, and 2595 with no bacterial growth (78.8%) and missing relevant data (6.9%), who were excluded from the study. The baseline characteristics and comorbidities of the 432 patients are summarized in Table 1. The mean age of our study population was 66.5 years ± 10.8 with 76.4% who were male. Patients were equally distributed between non-ICU (47.9%) and ICU (52.1%) admissions. Deaths among females were nearly double, compared to males (19.6% vs. 8.5%; *p* = 0.002). The most common co-morbidity was hypertension (33.8%), followed by nearly one-in-five patients with diabetes mellitus (25.5%), and corpulmonale (23%) (Table 1).

### 2.1. Bacterial Prevalence

**Figure 2:**
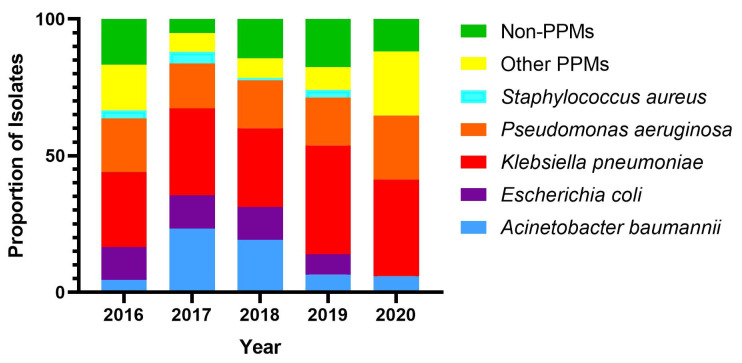
Bacterial prevalence was observed every year from 2016 to 2020. *Klebsiella pneumoniae* (32.4%) was the most prevalent, followed by *Pseudomonas aeruginosa* (17.8%), *Acinetobacter baumannii* (14.4%), *Escherichia coli* (10.4%), *Staphylococcus aureus* (2.5%), other PPMs (10.6%), and non-PPMs (11.8%). (Note: No *Escherichia coli* and *Staphylococcus aureus* were isolated in 2020, which was due to the COVID-19 pandemic which led to fewer patient admissions and in turn fewer culture-positive patients).

A total of 432 patients had bacterial AECOPD, in which *Klebsiella pneumoniae* (32.4%) was the most frequent organism isolated, followed by *Pseudomonas aeruginosa* (17.8%), *Acinetobacter baumannii* (14.4%), *Escherichia coli* (10.4%) and *Staphylococcus aureus* (2.5%), which together accounted for 77.5% of the total isolates. The remaining 22.5% of the isolates were other PPMs (10.6%), and non-PPMs (11.8%). Males had a higher frequency of infection with PPMs (90%) than females (82.4%) (*p* = 0.036). Over the five years, the number of *Acinetobacter baumannii* and *Escherichia coli* isolations decreased while the number of *Klebsiella pneumoniae* and *Pseudomonas aeruginosa* isolations increased (Figure 2).

### 2.2. Trends in Proportion of the Drug Resistance

**Figure 3:**
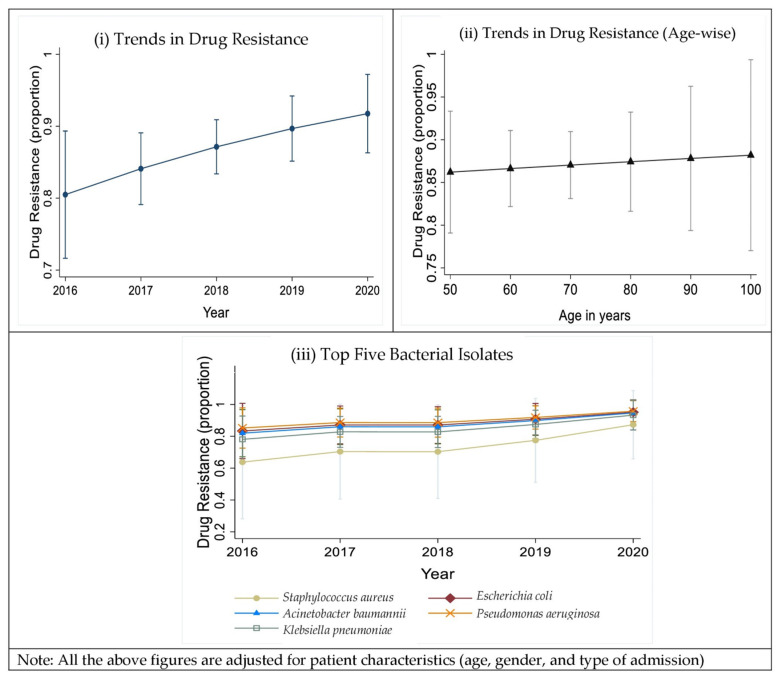
Trends in the overall drug resistance (DR, MDR, XDR), a gradual increase in the prevalence of drug resistance was observed, from the year 2016 to 2020 (**i**); Trends in the overall drug resistance (DR, MDR, XDR) adjusted with age (**ii**); Trends in the overall drug resistance of the top five bacterial organisms (*Acinetobacter baumannii*, *Escherichia coli*, *Klebsiella pneumoniae*, *Pseudomonas aeruginosa*, and *Staphylococcus aureus*, from the year 2016 to 2020 (**iii**). (Note: Proportion 0.1 = 10%, 1 = 100%; No *Escherichia coli* and *Staphylococcus aureus* were isolated in 2020, which was due to the COVID-19 pandemic, which led to fewer patient admissions and in turn fewer culture-positive patients).

A gradual increase in the prevalence of drug resistance was observed, from 2016 to 2020 (Figure 3i). Upon adjusting for the patient characteristics, an increasing trend in the overall drug resistance frequency was observed. The probability of drug resistance increased from 0.85 to 0.88 with increasing age (Figure 3ii). A significant increase in the proportion of drug resistance in *Pseudomonas aeruginosa* [RiskRatio (RiR) = 1.06; 95% CI (1.06, 1.67)], *Acinetobacter baumannii* [RiR = 1.05; 95% CI (1.05, 1.59)], *Escherichia coli* [RiR = 1.06; 95% CI (1.06, 1.06)] isolates was observed (Figure 3iii).

### 2.3. Trends in the Proportion of the Drug Resistance Isolates, Based on Sex and Type of Ward Admission

**Figure 4:**
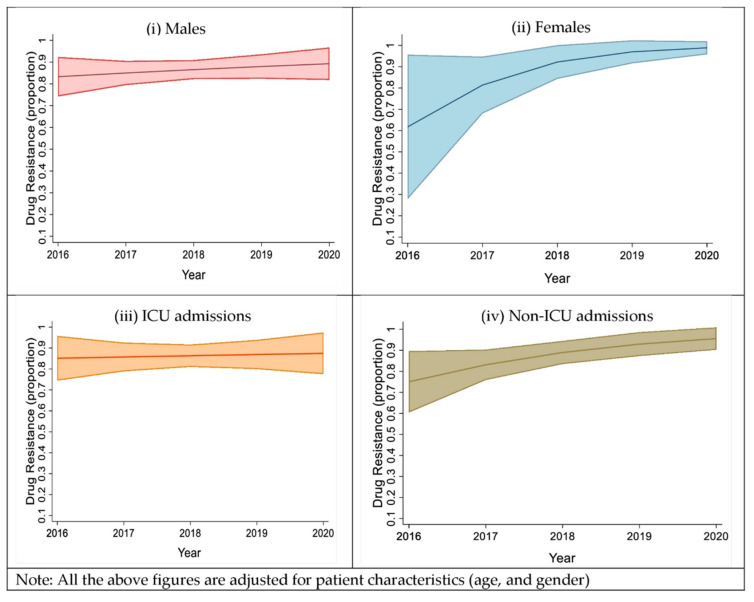
(**i**) shows the overall drug resistance in the top five organisms isolated from males, from the year 2016 to 2020; (**ii**) shows the overall drug resistance in the top five organisms isolated from females, over five years, a steep increase in the overall drug resistance was observed; (**iii**) shows the overall drug resistance in patients admitted to the ICU, from the year 2016 to 2020, and (**iv**) shows the overall drug resistance in patients admitted to non-ICU wards, from the year 2016 to 2020 and a gradual increase in the drug resistance was observed. (Note: Proportion 0.1 = 10%, 1 = 100%).

While the time trends showed a wide variation in the frequency of overall drug resistance among males, we observed a steep increase in the overall drug resistance, from 2016 to 2020, among females. This steep increase is mainly attributed to the increase in drug resistance among *Klebsiella pneumoniae* and *Pseudomonas aeruginosa.* We observed that the prevalence of drug resistance among males over five years increased from 82.5% to 89.5% and the prevalence of drug resistance among females over five years, increased from 62.5% to 99.5% (Figure 4i,ii). We further analyzed the prevalence of drug resistance among patients admitted to non-ICU wards over five years, compared to ICU admissions, and found that it increased from 74% to 95%. No interaction was observed between the type of ward admitted to and sex, but a high probability of drug resistance was observed for the top five isolates, mainly with the non-ICU admissions [aRiR = 1.20 95% CI (1.02, 1.41)] among both males and females (Figure 4iii,iv).

### 2.4. Trends in the Proportion of Multi-Drug Resistant Isolates

**Figure 5:**
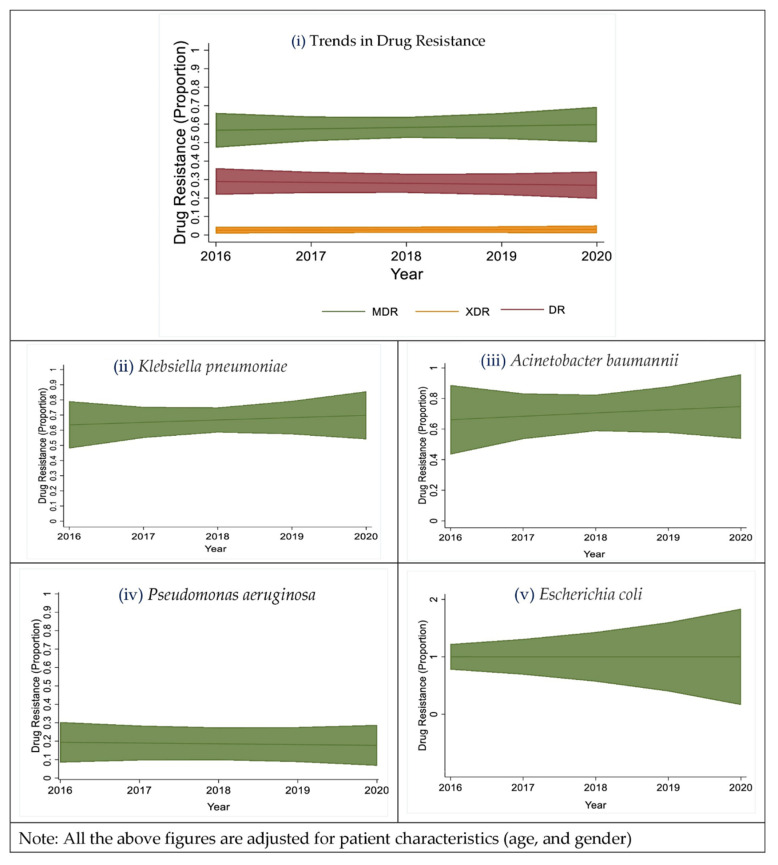
(**i**) shows the trends in single-drug resistance, multi-drug resistance, and extreme drug-resistance in the top five bacterial organisms isolated, from the year 2016 to 2020; (**ii**) shows the trends in the multi-drug drug resistance in *Klebsiella pneumoniae* isolates and the constant MDR organisms were isolated every year, from 2016 to 2020; (**iii**), shows the trends in the multi-drug drug resistance in *Acinetobacter baumannii* isolates, from the year 2016 to 2020; (**iv**) shows the trends in the multi-drug drug resistance in *Pseudomonas aeruginosa* isolates, from the year 2016 to 2020; and (**v**) shows the trends in the multi-drug drug resistance in *Escherichia coli* isolates, from the year 2016 to 2019; (Note: Proportion 0.1 = 10%, 1 = 100%; No *Escherichia coli* were isolated in 2020, which was due to the COVID-19 pandemic which led to fewer patient admissions and in turn fewer culture positive patients).

Over the five years, an increasing trend in drug resistance was observed among all of the organisms (except for *Staphylococcus aureus* as it had a very small number of isolates). Of the top five organisms which accounted for 77.5% of the total isolates, 57.6% were MDR, 26% were drug-resistant, 3.6% were XDR, and 12.8% were sensitive to all the drugs tested (Figure 5i). A gradual increase in MDR *Klebsiella pneumoniae* isolates was observed, from 2016 to 2020, adjusted for the patient characteristics (Figure 5ii). In 2016, more than 60% of *Acinetobacter baumannii* isolates were susceptible to all tested drugs, but a gradual rise in the frequency of MDR isolates was observed from 2016 to 2020, after adjusting for the patient characteristics (Figure 5iii). In 2016, more than 30% of *Pseudomonas aeruginosa* isolates were XDR, but a decrease in the XDR and an increase in the MDR *Pseudomonas aeruginosa* isolates was observed, from 2016 to 2020, adjusted for the patient characteristics (Figure 5iv). Every year, more than 80% of the *Escherichia coli* isolates were MDR, but no *Escherichia coli* were isolated in 2020 (which was due to the COVID-19 pandemic, which led to fewer patient admissions and in turn fewer culture-positive patients), adjusted for the patient characteristics (Figure 5v). All 11 isolates of *Staphylococcus aureus* over the five years were drug-resistant, of which one was a Methicillin-Resistant *Staphylococcus aureus* (MRSA). No *Staphylococcus aureus* was isolated in 2020 (which was due to the COVID-19 pandemic, which led to fewer patient admissions and in turn fewer culture-positive patients).

### 2.5. Trends of the Resistance for Different Drug Classes

**Figure 6:**
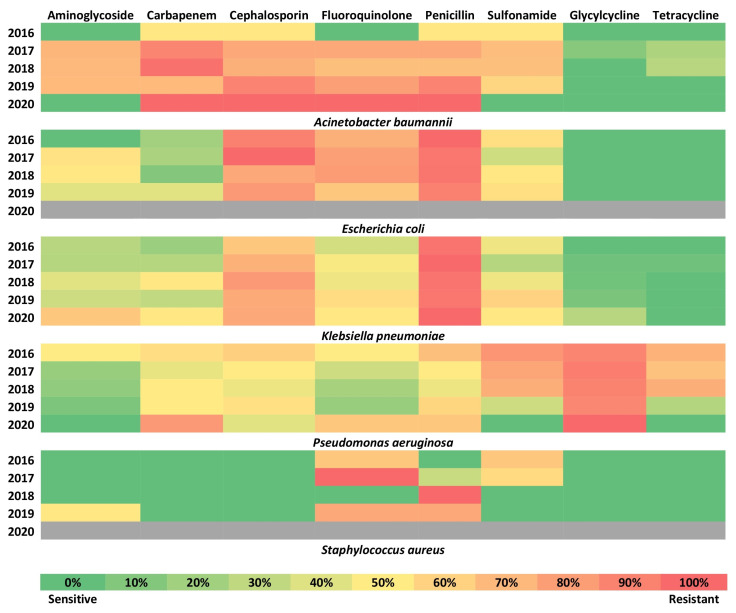
Trends of the isolates (*Acinetobacter baumannii*, *Escherichia coli*, *Klebsiella pneumoniae*, *Pseudomonas aeruginosa*, and *Staphylococcus aureus*) resistant to different antibiotic classes. Glycylcycline remained the most sensitive drug over the years for *Acinetobacter baumannii*. Glycylcycline and tetracycline remained the most sensitive drugs over the years for *Escherichia coli*. Tetracycline remained the most sensitive drug over the years for *Klebsiella pneumoniae*. Aminoglycosides were the best class of drugs over the years for *Pseudomonas aeruginosa.* (Note: No *Escherichia coli* and *Staphylococcus aureus* were isolated in 2020 which was due to the COVID-19 pandemic which led to fewer patient admissions and in turn fewer culture-positive patients).

Figure 6 shows the trends in the drug resistance over time for the five common organisms for different drug classes. Tetracycline and glycylcycline remained the most sensitive classes of drugs over the years for *Klebsiella pneumoniae*, *Acinetobacter baumannii*, and *Escherichia coli.* Penicillins, as a class, were found to have near-complete drug resistance for all the top five organisms. Aminoglycosides were the best class of drugs over the years for *Pseudomonas aeruginosa*. Fluoroquinolone was the second-best class of drugs for *Pseudomonas aeruginosa*. The third-best class of drugs was carbapenem for *Escherichia coli* and *Klebsiella pneumoniae*. Sulfonamide was the third-best class of drugs for *Acinetobacter baumannii* and *Pseudomonas aeruginosa*. The fourth-best class of drugs was aminoglycosides for *Acinetobacter baumannii*, *Escherichia coli*, and *Klebsiella pneumoniae* (Figure 6).

### 2.6. Mortality

A univariate Cox proportional hazard regression analysis to investigate mortality, showed that females had a significantly higher mortality risk. In a multivariate Cox proportional hazard regression analysis, an infection with *Acinetobacter baumannii* and females was independently associated with mortality. *Acinetobacter baumannii*, the third most common organism after *Klebsiella pneumoniae* (32.4%), and *Pseudomonas aeruginosa* (17.8%) had 2.64 (95% CI: 1.08–6.43) higher odds of death, compared to *Klebsiella pneumoniae.* Following the adjustment for drug resistance (model 2), the odds of death increased to 2.88 (95% CI: 1.18–7.03) (Table 2).

## 3. Discussion

Our study found that *Klebsiella pneumoniae* was the most frequent organism isolated, followed by *Pseudomonas aeruginosa*, *Acinetobacter baumannii*, *Escherichia coli*, and *Staphylococcus aureus*, among patients with AECOPD. Of major concern, over five years, of the top five bacterial species that accounted for 77.5% of all isolates, 57.6% were MDR, 26% SDR, 3.6% XDR, and only 12.8% were susceptible to all drugs tested. The mortality rate among AECOPD patients infected with drug-resistant bacteria was 12.7%, compared to 4.7% among drug-sensitive patients. Females had a significantly higher risk of mortality, as compared to males (20.5% in females vs. 9.2% in males). Females also had higher death rates with (21.2%) and without (14.3%) drug resistance, compared to males with (10.2%) and without (2.8%) drug resistance. Both males and females had an increasing trend of drug resistance, however, females had a steeper increase in drug resistance over the five years. Eighty-five percent of isolates from ICU were drug-resistant in 2016, which marginally increased over five years. In contrast, drug resistance in isolates from non-ICU was 75% in 2016 which increased to more than 90% over the five years.

Bacterial superinfections cause substantial morbidity and mortality among AECOPD patients worldwide. Bacteria are isolated from sputum in 40% to 60% of AECOPD patients [6]. The bacterial load, diversity, virulence, and pathogenicity increase during COPD and are worsened by the host factors, such as increasing age, impairment of lung function, comorbid conditions, and smoking [11]. Other factors that could contribute to COPD exacerbations include infection from hospital flora that are drug-resistant or other co-infecting species, inflammatory response, hypoxia, or nutrient deficiency [12]. Once a COPD patient develops a bacterial exacerbation, there is an increased risk of disease progression to respiratory failure, ICU admissions, longer hospital stays, and death [11,12].

Globally, the top four common bacteria causing AECOPD include *Haemophilus influenzae*, *Pseudomonas aeruginosa*, *Streptococcus pneumoniae*, and *Moraxella catarrhalis***.** A review conducted by Rodrigo-Troyano et al. found that *Klebsiella pneumoniae* (in Asia) and *Pseudomonas aeruginosa* (in Europe) were the most common Gram-negative bacteria isolated in AECOPD patients [13]. Previous studies conducted globally, demonstrated a significant variation in the prevalence of pathogens in AECOPD, compared to Indian studies in which the common organisms found in different studies were *Klebsiella pneumoniae*, *Streptococcus pneumoniae*, *Pseudomonas aeruginosa*, and *Acinetobacter baumannii* [14,15,16]. Furthermore, wide variations in the global prevalence of the different bacteria were observed: *Klebsiella pneumoniae* (1% to 58%), *Pseudomonas aeruginosa* (1.2% to 29.1%), *Acinetobacter baumannii* (0.8% to 14%), *Escherichia coli* (0.4% to 11%), and *Staphylococcus aureus* (0.1% to 26%). The high isolation rates of *Klebsiella pneumoniae*, *Pseudomonas aeruginosa*, and *Acinetobacter baumannii* in the present study are in contrast to the studies from regions outside of India, while they resemble those reported in other Indian studies. It is unclear whether this is related to the differences in broad-spectrum antibiotic use, exposure to hospital flora during their visits to the hospital, or the geographical variations in bacterial distributions.

*Klebsiella pneumoniae* as a predominant bacterium causing AECOPD, is seen mostly in Asian and Indian studies. Globally, the drug resistance in *Klebsiella pneumoniae,* especially to carbapenems, is increasing, which poses a serious threat to patients because of its association with increased fatality. In our study, the carbapenem resistance of *Klebsiella pneumoniae* increased from 11.1% to 33.3%, between 2016 and 2020. In India, *Pseudomonas aeruginosa* was usually found to be the second most common bacteria causing AECOPD after *Klebsiella pneumoniae* but in some studies, it is the most predominant [17,18]. Globally, *Acinetobacter baumannii* and *Escherichia coli* are, in general, not commonly associated with AECOPD [19,20]. In India, *Acinetobacter baumannii* was prominent in one prospective study among ICU patients [15] with AECOPD, compared to other Indian studies [16,21].

In our study, the highest susceptibility of the AECOPD-associated bacteria, was to tetracycline and glycylcycline (more than 80% of *Acinetobacter baumannii*, *Escherichia coli*, *Klebsiella pneumoniae*, and *Staphylococcus aureus* isolates). Few Indian studies that have evaluated tigecycline, have observed a sensitivity rate of 66.7% to 100%. Globally, for non-*Pseudomonas aeruginosa* infections, tigecycline has not been commonly used. Among tetracyclines, doxycycline has been used with a sensitivity rate of 12.5% to 77.8% [22,23]. In our study, *Pseudomonas aeruginosa* was intrinsically resistant to tigecycline (90% of isolates) and was most sensitive to aminoglycosides (88.3). Similarly, aminoglycosides (50% to 100%) and fluoroquinolones (47.4% to 100%) were the most appropriate antibiotics for pseudomonal infections, globally [24,25].

We observed an increasing trend of the drug-resistant infections in females with AECOPD over the last five years, the cause of which needs to be ascertained. Several reports have observed that females have a greater risk of antimicrobial resistance (AMR), due to both biological and sociocultural issues [26,27,28,29]. We have not evaluated these issues in our patients, but the possible reasons include biological factors, type of employment, excessive home-based care work, and exposure to live stocks (dairy, poultry) where antibiotics are rampantly misused [30] and limited access to healthcare and therefore self-medication with broad-spectrum antibiotics [26]. They also have a higher exposure to animals carrying resistant bacteria, as they are involved in performing menial tasks in agriculture and animal husbandry [28]. Gender differences in antibiotic prescriptions by doctors, due to a lack of training and gender-bias, increase female antibiotic usage [26] where females are 27% more likely to receive an antibiotic prescription, compared to men [31]. Females also have a higher exposure to antibiotics during pregnancy, childbirth, and abortion [26].

The highest consumer of antibiotics in the world is India [32]. According to the annual report of the Antimicrobial Resistance Research and Surveillance Network of ICMR. The most commonly isolated bacteria were *Escherichia coli*, followed by *Klebsiella pneumoniae*, *Pseudomonas aeruginosa*, *Acinetobacter baumannii*, and *Staphylococcus aureus.* In India, a sustained rise in antimicrobial resistance has been observed [33]. Globally, infections with antimicrobial-resistant isolates contribute to 700,000 deaths, annually [34]. The mortality rates, due to age-standardized infectious illness in India are among the highest in South Asia, and the levels of antibiotic resistance are concerning [35]. In the case of hospital-acquired infections, carbapenem resistance *Klebsiella* spp. are commonly seen with a case fatality rate of about 50% [36] and when this *Klebsiella* spp. have polymyxin resistance, the case fatality rate is approximately 70% [37].

Globally, the mortality due to bacterial infections in AECOPD patients, varied from 2.32% to 50% [19,20]. In India, the mortality, due to bacterial infections in AECOPD patients varied, from around 3.1 to 15% [15,18]. We observed mortality rates of 11.1%, which was significantly higher in females (19.6%), as compared to males (8.5%). Prescott et al., and Ringbaek et al., in their study, observed that the excess loss of lung function associated with smoking was greater in females than in males and after adjusting for smoking, females had a higher risk of hospital admission, associated co-morbidities, and death due to COPD, than males [38,39]. The reasons for the higher mortality in females are not clear. Several hypotheses have been suggested; Females are more prone to COPD exacerbations and experience impaired quality of life at an earlier age of onset, than men [40]. Females have smaller airways than males for the same lung volume, which might lead to a greater concentration of tobacco smoke per unit area of the small airway surface [41]. Cigarette smoke might metabolize differently in females because of the sex differences in the expression of cytochrome p450 enzymes. Tam et al. observed that female mice developed a higher peripheral airway obstruction and small airway remodeling than male mice, who developed predominant emphysema, but ovariectomy produced the same pattern as in male mice, indicating that female sex hormones could be responsible for these differences. They also observed that the estrogen receptor-α (ERα) blocker tamoxifen mimicked the effects of ovariectomy, indicating additional evidence that estrogen accounts for these sex differences, in response to chronic smoking [42]. Not all of the studies observed a higher mortality among females. While few studies found that all-cause and respiratory mortality are usually higher in males than females with AECOPD, others observed no difference between both sexes [43,44]. One of the other unique features of our study population, in comparison to the western population, merits consideration. Almost all of the men had tobacco smoke-induced AECOPD and almost all of the females had biomass smoke-induced AECOPD. Along with tobacco smoking, biomass smoke exposure is a major risk factor for the development of AECOPD in LMIC countries, that has different clinical characteristics, compared to tobacco smoke AECOPD and most of the biomass-exposure AECOPD patients are women and have worse symptoms and quality of life, compared to tobacco smoke AECOPD [45]. While tobacco smoking AECOPD is linked to greater emphysema, exposure to biomass smoke is linked to a higher incidence of bronchial thickening, bronchiectasis, small airway disease, airway fibrosis, pulmonary hypertension, and arteriolar intimal thickening [46].

We observed high levels of drug resistance in AECOPD patients admitted to both the ICU (87.8%) and non-ICU (86.5%). While the high rates of drug resistance in ICU are expected, almost similar rates of drug resistance in non-ICU settings were unexpected and need urgent investigation. Possible reasons include, that AECOPD patients are prescribed three times more antibiotics than the general public [47], antibiotic misuse with many general practitioners prescribing broad-spectrum antibiotics for trivial infections or viral infections [48], a higher risk of developing bacterial infections, due to impaired host defenses [49], thus prompting frequent antibiotic use, AECOPD lungs, are more conducive to form biofilms which provide a physical barrier to the antibiotic infiltration and facilitate the development of drug resistance [50].

### Strength and Limitations

To our knowledge, this is the first study examining the trends in drug resistance among infections of AECOPD patients across gender, ICU, and non-ICU admissions in India. We used advanced statistical methods, including GLM and GEE, to identify trends after adjusting for the confounders. This is one of the few comprehensive studies conducted in LMICs that have evaluated the impact of gender on AECOPD-associated mortality. The limitation of the study includes a lack of generalizability, due to data collection happening on one site. Information on the frequency and type of antibiotic used over the last few years was not available for many cases and could not be used in the analysis. As it is a retrospective study, data on patient readmissions and previous admissions were also not collected. No information on exacerbation, caused by viruses and fungi or mixed organisms was collected.

## 4. Materials and Methods

### 4.1. Study Population

This retrospective study was carried out with data collected from patients with AECOPD, admitted to a university-affiliated 1800-bed tertiary care hospital catering to the healthcare needs of patients from the Mysore District, JSS Medical College & Hospital, India, between January 2016 and December 2020. The hospital information system (HIS) uses the ICD-10 coding system and all subjects with AECOPD (J44.1 and J44.9) were screened for demographic characteristics (age and sex), comorbidities, length of hospital stay (LOS), and admission to ICU, or non-ICU, during the study period, and mortality. The information on the bacterial pathogen species and their drug susceptibility patterns were collected. We identified 3027 COPD patients who were admitted to the tertiary care hospital, from January 2016 to December 2020, which constituted the Mysuru COPD (MYCO) Cohort. Four hundred and thirty-two patients had bacterial infections and were included in the study. The COPD patients without bacterial exacerbations were excluded. Any additional secondary diagnoses were defined, according to the ICD10 coding system.

### 4.2. Study Definitions

AECOPD was defined as the acute worsening of respiratory symptoms (e.g., increase in cough or quantity of sputum production or breathlessness; or increase in the purulence of sputum) that result in additional therapy [51]. Charlson’s co-morbidity index, which is a composite risk of all associated co-morbidities where higher scores predict a higher risk of mortality or higher resource use, was calculated [52]. The definitions we used in the study for multidrug-resistant (MDR), extensively drug-resistant (XDR), and pan-drug-resistant (PDR) pathogenic bacteria, were based on the classification proposed by Magiorakos et al. [53]. In our study, we defined a bacterial strain as drug-resistant if it was not susceptible to one agent in one or more antimicrobial drug classes. Single drug-resistant (SDR) was defined as strains non-susceptible to one agent in one antimicrobial class. MDR was defined as those strains non-susceptible to at least one agent in three or more antimicrobial classes. XDR was defined as strains non-susceptible to at least one agent in all but two or fewer antimicrobial classes, and if the strains were non-susceptible to all the antimicrobial agents used, they were defined as PDR.

### 4.3. Microbiological Study

Sputum samples that were received in the Microbiology laboratory for routine diagnosis were included in the study. The samples were processed, according to the departmental standard operating procedures. In brief, the samples that were received were subjected to gram staining to assess the quality of the sputum sample, and those samples with Bartlet scores that are 0, −1, and −2 were excluded from the study. Sputum samples with Bartlet scores of +1, and +2 were included and were inoculated onto Blood agar and Mac Conkey agar for the isolation of the pathogens, and the media was incubated at 37 degrees Celsius overnight. The growth was further identified and antibiotic susceptibility testing of the isolates was determined by the Vitek^®^ 2 system (BioMérieux, Lyon, France, 4.4) All of the clinical and demographic data of the subjects included in the study was collected from HIS for screening. Based on the organism isolated, we classified the bacteria into potentially pathogenic microorganisms (PPMs) and non-PPMs, as described by Cabello et al. [54].

The microorganisms recognized as agents causing respiratory infections were classified as PPMs, whether or not they belonged to the gastrointestinal or oropharyngeal flora. Organisms classified as PPMs consisted of *Klebsiella pneumoniae*, *Pseudomonas aeruginosa*, *Acinetobacter baumannii*, *Escherichia coli*, *Staphylococcus aureus*, *Acinetobacter junii*, *Acinetobacter lwoffii*, *Acinetobacter ursingii*, *Citrobacter freundii*, *Citrobacter koseri*, *Citrobacter species*, *Enterobacter aerogenes*, *Enterobacter cloacae complex*, *Haemophilus influenzae*, *Klebsiella oxytoca*, *Proteus mirabilis*, *Pseudomonas fluorescens*, *Pseudomonas putida*, *Serratia marcescens*, *Stenotrophomonas maltophilia*, and *Streptococcus pneumoniae*.

The microorganisms that are not usually involved in respiratory infections in non-immune deficient patients were classified as non-PPMs belonging to gastrointestinal or oropharyngeal flora. Organisms classified as non-PPMs included *Aeromonas species*, *Alpha Haemolytic Streptococci*, *Beta Haemolytic Streptococci*, *Burkholderia cepacia*, *Coagulase-negative Staphylococcus*, *Group A Streptococci*, *Sphingomonas paucimobilis*, *Staphylococcus capitis*, *Staphylococcus epidermidis*, *Staphylococcus haemolyticus*, *Staphylococcus hominis ssp hominis*, *Staphylococcus saprophyticus*, *Staphylococcus sciuri*, *Staphylococcus warneri*, and *Streptococcus species*.

### 4.4. Antibiotic Susceptibility

From January 2016 to December 2020, a total of 432 bacteria isolated, were subjected to antibiotic susceptibility testing, which includes aminoglycosides (amikacin and gentamicin), carbapenems (doripenem, ertapenem, imipenem, and meropenem), other cephalosporins (cefepime, cefoperazone/sulbactam, cefotaxime, cefoxitin, ceftazidime, ceftriaxone, and cefuroxime), fluoroquinolones (ciprofloxacin, gatifloxacin, levofloxacin, and, norfloxacin) for gram-negative bacteria and penicillin’s (amoxicillin-clavulanic acid, ampicillin, oxacillin, penicillin-g, piperacillin/tazobactam, and ticarcillin/clavulanic acid), macrolides (erythromycin), tetracycline (minocycline), glycylcycline (tigecycline), glycopeptides (vancomycin and teicoplanin), sulfonamides (trimethoprim/sulfamethoxazole), lipopeptide (colistin), phenicol (chloramphenicol), nitrofurantoin, and oxazolidinones (linezolid) for gram-positive bacteria.

### 4.5. Inclusion and Exclusion Criteria

The eligible subjects were identified by the ICD-10 codes J44.1 and J44.9. Male and female subjects ≥40 years of age, diagnosed with AECOPD, who tested sputum culture-positive, were included in the study. Other respiratory samples, subjects with no bacterial growth, and relevant missing data, were excluded from the study.

### 4.6. Statistical Analysis

The descriptive data were presented as the mean (±standard deviation) of continuous variables and as frequencies of discrete variables. The student *t*-test for continuous variables and the chi-squared test for the categorical variables were compared through a univariate analysis to assess the statistical significance. Statistical analyses were performed with SPSS software v.22 (IBM, Corp., Armonk, NY, USA) and the Stata 16 version (StataCorp., College Station, TX, USA). All statistical tests were two-tailed, and the factors were considered statistically significant at *p* < 0.05 and were included in the generalized linear model (GLM) and used a generalized estimating equation (GEE) if they were found to be statistically significant on a univariate analysis. Figures were generated with Microsoft Excel^®^ and GraphPad Prism software (GraphPad Software Inc., San Diego, CA, USA). The antibiotic activity of the bacterial isolates was recorded as (susceptible = 0, resistant = 1) responses, with other covariates (time in years, age, sex, and comorbidities) as the explanatory variables. The proportion of each type of antibiotic resistance was summarized as drug-sensitive, SDR, MDR, and XDR. We adjusted the frequencies of the patient’s characteristics (age, gender, comorbidities) and type of admissions to understand the relationship of the GLM with binomial error, and logit link. We further used a GEE to explore the linear trends between the rate of antibiotic resistance and the top five bacterial species isolates over a time span of five years (2016–2020). In this study, we used the GEE model with the binomial link, and *p*-values, less than 0.05 were considered statistically significant. The GEE was used to account for the repeated measure and scanty data. As such, we analyzed the five most common bacterial species isolates that accounted for approximately 80 percent of any or all drug resistance. We looked for interactions between admissions to different hospital units (i.e., ICU and non-ICU), yearly. The multivariate Cox regression analysis was used to identify the independent risk factors associated with mortality.

### 4.7. Ethics Approval

This study was approved by the Institutional Ethics Committee of JSS Medical College, Mysuru (Approval number: JSSMC/IEC/18.02.2022/24NCT/2021-22). Informed consent was waived because this was a retrospective study.

## 5. Conclusions

A high proportion of COPD bacterial exacerbations were found to be drug-resistant both in the ICU and non-ICU settings. *Klebsiella pneumoniae* was the most common organism causing bacterial AECOPD, followed by *Pseudomonas aeruginosa* and *Acinetobacter baumannii*. Over five years, an increasing trend in drug resistance was observed in AECOPD hospital admissions. Female sex and *Acinetobacter baumannii* were independent risk factors for mortality and drug resistance. Antimicrobial stewardship with the judicious use of antimicrobial agents is essential to prevent the emergence of resistant, MDR, XDR, and PDR bacteria in AECOPD patients.

## Figures and Tables

**Table 1:** Describes the clinical, demographic, microbiologic characteristics, survival of the study subjects, and the test of significance between the male and female subjects with AECOPD.

	Total (n = 432)	Male (n = 330)	Female (n = 102)	*p*-Value
Age (Mean ± SD)	66 (60 to 75)	66.8 ± 10.7	65.6 ± 11.3	0.413 *
Length of hospital stay (Median (IQR)	6.0 (4.0 to 10)	6.0 (4.0 to 10)	7.0 (4.0 to 9.75)	0.668 *
Non-ICU (n, %)	207 (47.9)	162 (49.1)	45 (44.1)	0.380 ^†^
ICU (n, %)	225 (52.1)	168 (50.9)	57 (55.9)	
Alive (n, %)	384 (88.9)	302 (91.5)	82 (80.4)	0.002 ^†^
Dead (n, %)	48 (11.1)	28 (8.5)	20 (19.6)	
Potentially pathogenic microorganisms (PPMs)				
Yes (n, %)	381 (88.2)	297 (90)	84 (82.4)	0.036 ^†^
No (n, %)	51 (11.8)	33 (10)	18 (17.6)	
Drug resistance				
Yes (n, %)	292 (87.2%)	226 (86.3%)	66 (90.4%)	
No (n, %)	43 (12.8%)	36 (13.7%)	7 (9.6%)	0.348 ^†^
Charlson’s comorbidity index (CCI) (Median (IQR))	4.0 (3.0 to 5.0)	4.0 (3.0 to 5.0)	4.0 (3.0 to 5.0)	0.289 *
Comorbidities				
Diabetes mellitus (n, %)	110 (25.5)	78 (23.6)	32 (31.4)	0.117 ^†^
Heart disease (n, %)	64 (14.8)	53 (16.1)	11 (10.8)	0.190 ^†^
Kidney disease (n, %)	50 (11.6)	42 (12.7)	8 (7.8)	0.178 ^†^
Liver disease (n, %)	17 (3.9)	16 (4.8)	1 (1)	0.079 ^†^
Corpulmonale (n, %)	99 (22.9)	63 (19.1)	36 (35.3)	<0.001 ^†^
Hypertension (n, %)	146 (33.8)	99 (30)	47 (46.1)	0.003 ^†^
Obesity (n, %)	24 (5.6)	13 (3.9)	11 (10.8)	0.008 ^†^
Pulmonary Hypertension (n, %)	89 (20.6)	67 (20.3)	22 (21.6)	0.782 ^†^
Sepsis (n, %)	33 (7.6)	25 (7.6)	8 (7.8)	0.929 ^†^

AECOPD: Acute exacerbation chronic obstructive pulmonary disease. * Kruskal–Wallis test. ^†^ Pearson Chi sq test. Significant = *p* < 0.05.

**Table 2:** Univariate and multivariate Cox regression analysis of the risk factors associated with mortality.

Dependent	Model 1		Model 2	
	HR (Univariable)	HR (Multivariable)	HR (Univariable)	HR (Multivariable)
Bacterial Species				
*Klebsiella pneumoniae *	Reference	Reference	Reference	Reference
*Pseudomonas aeruginosa *	1.60 (0.66–3.88)	1.63 (0.67–3.99)	1.60 (0.66–3.88)	1.94 (0.77–4.92)
*Acinetobacter baumannii *	2.27 (0.96–5.35)	2.64 (1.08–6.43) *	2.27 (0.96–5.35)	2.88 (1.18–7.03) *
*Escherichia coli *	1.77 (0.60–5.18)	1.82 (0.61–5.40)	1.77 (0.60–5.18)	1.79 (0.60–5.32)
*Staphylococcus aureus *	2.43 (0.53–11.11)	2.50 (0.54–11.62)	2.43 (0.53–11.11	2.50 (0.54–11.60)
Gender				
Male	Reference	Reference	Reference	Reference
Female	2.62 (1.35–5.08) *	2.89 (1.47–5.70) *	2.62 (1.35–5.08) *	2.71 (1.36–5.37) *
Age in years (Mean (SD))	1.02 (0.99–1.05)	0.99 (0.95–1.04)	1.02 (0.99–1.05)	0.99 (0.94–1.04)
CCI (Mean (SD))	1.19 (0.96–1.48)	1.31 (0.95–1.80)	1.19 (0.96–1.48)	1.33 (0.97–1.83)
Drug resistance				
No	-	-	Reference	Reference
Yes	-	-	2.01 (0.48–8.37)	2.36 (0.53–10.51)

Model 1: includes bacterial species, gender, age, and CCI. Model 2 includes bacterial species, gender, age, CCI, and drug resistance; * = *p* < 0.05, HR: Hazards ratio, CCI: Charlson’s comorbidity index, SD: Standard deviation.

## Data Availability

All data generated or analyzed during this study are included in this article.
